# Cases from My Note-book

**Published:** 1883-04

**Authors:** Julius Schmitt

**Affiliations:** Rochester, N. Y.


### CLINICAL BUREAU.

---

### CASES FROM MY NOTE-BOOK.

JULIUS SCHMITT, M. D., ROCHESTER, N. Y.

CASE III. March 29th, 1882.—G. M., 42 years old, German, grocer; dark complexion, robust, very active business man—keeps a saloon besides his grocery, which, of course, makes him partake of spirituous drinks every day, but is no drunkard. Complains since several weeks of a steady pressure in both regions of kidneys, with pains going along both ureters to bladder, especially when walking and sitting; not when lying down at night. Frequent micturition day and night. Urine very light-colored. He can resist the frequent desire when actively engaged in his business. Feels so sleepy between 8 and 9 P. M.; lasts for one and a half hours, then it passes off. Thirsty; no appetite.

He also noticed recently an *undulating pain between right eyeball and roof of orbit*. Sexual weakness. (The symptom in italics, which was the latest, and certainly a very peculiar one, was searched for at once and found on page 78 of Berridge's excellent *Repertory of the Eye*, under Platinum.) After carefully comparing its provings according to Allen's *Materia Medica* with the symptoms of our patient, it was prescribed as follows: ℞ Platinum$^{30}$, eight powders night and morning.

April 3d.—Pressure in both kidneys better. Micturition much less frequent. Once in a while some pains down the ureters. Sleepiness at 8 and 9 P. M. is better. Yesterday he had *considerable pain in left eye of the same character and in the same place* as he had experienced in right eye, from which it has disappeared entirely. (This is the first time that this pain has been produced on left side.)

Since yesterday feels very bloated and bowels have not moved. He felt so uneasy that he took a dose of Sulphate of Magnesia, which operated on him once this morning. Gave him a good lecture and ℞ Sacch. lact.

April 6th.—Feels good in every respect, but bowels bother him a good deal; movements very hard, and there is a continuous, unsuccessful urging down to rectum, which, however, seems to be hermetically closed.

Rectum continues to pain. Bad taste in mouth. ℞ Nux vomica$^{cm}$, four powders, night and noon.

April 11th.—Reports stool every day, but had to start them by an enema. Bad taste in mouth, especially mornings; tongue coated light-yellowish. Back does not feel all right yet. A few days ago, while stooping, he felt a sudden stitch in back, which is repeated every time he gets into this position. Sometimes very hungry, so that he feels faint. All other symptoms gone. ℞ Sulphur$^{1000}$, one dose and placebo. Felt soon better and remains well ever since.

CASE IV. May 1st, 1882, Mathilde K., 7 years old, blonde, blue eyes; had, four weeks ago, pleuritis exsudativa dextra, which got apparently well under two doses of Belladonna$^{cm}$—at least parents failed to report until to-day, when I was called to see her again.

She complains of pain in right chest; worse when coughing; has to press her hand there. Cannot lie on painful side, which is also tender to touch. Right side of thorax is drawn in, so that the child walks leaning toward that side. Spinal column bent—convexity pointing to healthy side. Ribs turned up and almost overlapping each other, like shingles on a roof. Dullness on percussion of entire right lung. Auscultation shows no respiratory sounds at all, except high up under the shoulder-blade and in front in infra clavicular region, where there is bronchial breathing.

Very feverish to-day. Skin hot to touch, face, lips, and hands cyanotic. Difficult breathing. Short, dry cough, excites sometimes vomiting and suffocates her. *Bowels constipated;* if they move they are *very hard* and *smell very offensive.* Appetite not good, *desire for bread.* Urine is voided *only once a day*, and after *long straining* and in small quantity. *Strong-smelling perspiration* about head, neck, and on chest.

(Prognosis in cases like this, where the lung remains compressed after the pleuritic exudation has been absorbed, is very unfavorable, according to allopathic authorities. Patients will die of anasarca, caused by over-taxation of right heart and degeneration of kidneys or of consumption.)

Now let us see what pure Hahnemannian Homœopathy can do! ℞ Silicea$^{cm}$ (Swan), one dose, and Sacch. lact.

May 4th, 1882.—The same night following the above dose she had a very severe chill, followed by high fever, since then no fever; cough is looser. Bowels moved to-day, dry, reddish, and smelling badly. Urine is voided oftener and in greater quantity. She still asks for bread; all other symptoms the same. ℞ Sacch. lact.

May 14th.—Appetite is somewhat better; still there is the same desire for bread; bowels the same. She cannot bear any cold air; complains a good deal of chilliness. Cough very dry again. She says "there is something turning around in my stomach" (abdomen). Abdomen very tender to pressure; function of kidneys better. ℞ Silicea$^{cm}$ (Swan), one dose, and Sacch. lact.

May 25th.—The same night after receiving the dose of Silicea a very high fever set in, which lasted for two days. This was followed by a continuous improvement, without any further disturbance until the 1st of August, when I saw her the last time. There was then no symptom of disease left. The right lung had expanded to its normal size, and ausculation and percussion the same as in healthy side. Curvature of spine had entirely disappeared and the child was discharged as cured.

